# Identification of herbarium specimen sheet components from high‐resolution images using deep learning

**DOI:** 10.1002/ece3.10395

**Published:** 2023-08-14

**Authors:** Karen M. Thompson, Robert Turnbull, Emily Fitzgerald, Joanne L. Birch

**Affiliations:** ^1^ University of Melbourne Melbourne Victoria Australia

## Abstract

Advanced computer vision techniques hold the potential to mobilise vast quantities of biodiversity data by facilitating the rapid extraction of text‐ and trait‐based data from herbarium specimen digital images, and to increase the efficiency and accuracy of downstream data capture during digitisation. This investigation developed an object detection model using YOLOv5 and digitised collection images from the University of Melbourne Herbarium (MELU). The MELU‐trained ‘sheet‐component’ model—trained on 3371 annotated images, validated on 1000 annotated images, run using ‘large’ model type, at 640 pixels, for 200 epochs—successfully identified most of the 11 component types of the digital specimen images, with an overall model precision measure of 0.983, recall of 0.969 and moving average precision (mAP0.5–0.95) of 0.847. Specifically, ‘institutional’ and ‘annotation’ labels were predicted with mAP0.5–0.95 of 0.970 and 0.878 respectively. It was found that annotating at least 2000 images was required to train an adequate model, likely due to the heterogeneity of specimen sheets. The full model was then applied to selected specimens from nine global herbaria (*Biodiversity Data Journal*, 7, 2019), quantifying its generalisability: for example, the ‘institutional label’ was identified with mAP0.5–0.95 of between 0.68 and 0.89 across the various herbaria. Further detailed study demonstrated that starting with the MELU‐model weights and retraining for as few as 50 epochs on 30 additional annotated images was sufficient to enable the prediction of a previously unseen component. As many herbaria are resource‐constrained, the MELU‐trained ‘sheet‐component’ model weights are made available and application encouraged.

## Graphical abstract

An effective object detection model has been built to enable the automated segmentation of specimen images into their component parts and the isolation of text‐bearing labels from the herbarium specimen. This baseline model can increase the efficiency and accuracy of downstream data capture during digitisation of and data extraction from herbarium specimen images. Comprehensive testing of these models to specimens from global herbaria indicates the potential application of these methods and models for rapid extraction of biodiversity data from high‐resolution specimen images.

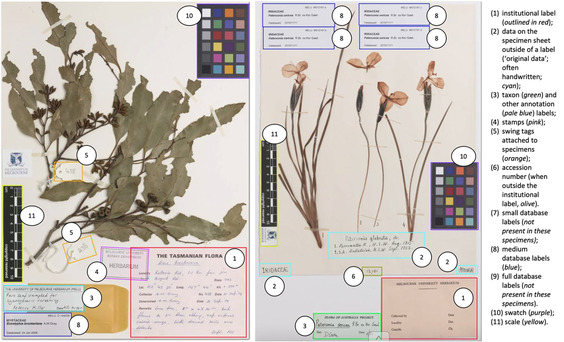

## 1 INTRODUCTION

There are approximately 3000 herbaria globally, collectively containing an estimated 350 million specimens (Thiers et al., 2016). Large‐scale digitisation projects, mobilising specimen‐associated data and generating digital specimen images, are underway in herbaria globally, to ensure specimen‐associated ecological, morphological and phenological data are accessible for use in integrative biodiversity research (Soltis, 2017). Advanced computer vision techniques hold the potential to overcome the significant bottleneck for data digitisation, that is the manual labour required for extraction of these data. These techniques are increasingly being used to extract text and trait‐based data from specimen images (Carranza‐Rojas et al., 2017; Ott et al., 2020; Triki et al., 2022; Younis et al., 2020). Greater understanding of the accuracy and efficiency of computer vision techniques as applied to different kinds of herbarium specimens is necessary to understand the potential application of these methods for data mobilisation.

**FIGURE 1:**
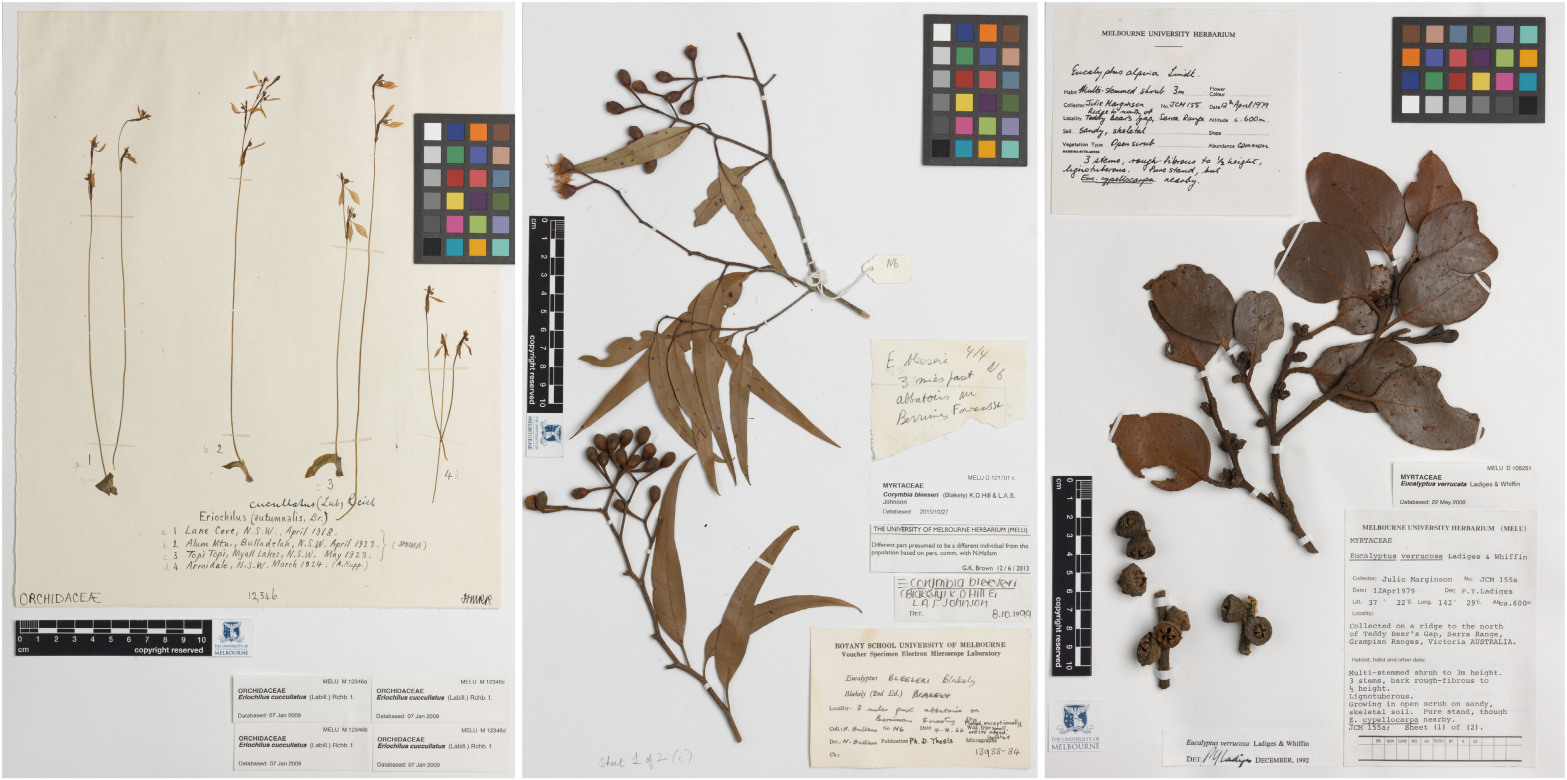
Examples of specimen sheet digital images from the Melbourne University Herbarium (MELU) (left) MELUM012346a–d (https://online.herbarium.unimelb.edu.au/collectionobject/MELUM012346a); (middle) MELUD121701c (https://online.herbarium.unimelb.edu.au/collectionobject/MELUD121701c); (right) MELUD105252a (https://online.herbarium.unimelb.edu.au/collectionobject/MELUD105252a).

Herbarium specimens and their associated collection data contain a wealth of biodiversity data; documenting morphological diversity, geographic distributions, biome or vegetation occupancy and flowering and fruiting periods of the taxon represented on the specimen, and how these may change over time. These typically dried pressed plant samples are secured to archival sheets, and are accompanied by label(s) on the sheet detailing collector, location and taxon and occasionally contain other elements such as stamps, handwritten notes (outside the label) and accession numbers (Figure 1). Large‐scale digitisation efforts are required in order to provide access to herbarium specimen‐associated data (Carranza‐Rojas et al., 2017) and to ensure these data are FAIR (findable, accessible, interoperable and reusable; Wilkinson et al., 2016). Critical to the success of the digitisation endeavour is an efficient, scalable, adaptable and cost‐effective workflow. An ‘object to image to data’ workflow, which involves the generation of a digital image of the specimen followed by the transcription of data from the digital image, is used in large‐scale digitisation initiatives such as that undertaken by the National Herbarium of New South Wales in Australia (Cox, 2022). The visibility of the specimen label data in the corresponding digital image ‘allows the data capture process to be undertaken remotely, both in distance and time’ (Haston et al., 2015, p. 116). Digitising enables creation of a ‘digital specimen’ (Nieva de la Hidalga et al., 2020): generating a digital image of each specimen sheet, manually transcribing some or all of the data present on the specimen label into a searchable database, and then sharing that information for reuse via online biodiversity repositories such as the Atlas of Living Australia (ALA; https://www.ala.org.au/), Global Biodiversity Information Facility (GBIF; https://www.gbif.org/) and iDigBio (https://www.idigbio.org/).

In recent years, research has focussed on optimising specific tasks *within* such digitisation workflows. Particularly evident is the desire to minimise or remove manual intervention, speed up the process, improve accuracy and reduce costs, particularly with respect to label data transcription (e.g. Granzow‐de la Cerda & Beach, 2010; Walton, Livermore, & Bánki, 2020; Walton, Livermore, Dillen, et al., 2020). Studies have tackled streamlining the imaging process (e.g. Sweeney et al., 2018; Tegelberg et al., 2014) and extending the use of digital images (e.g. Carranza‐Rojas et al., 2017; Corney et al., 2018; Triki et al., 2021; Unger et al., 2016; White et al., 2020). The task of interest here is that of harvesting label data from a specimen sheet digital image (SSDI). Important information is held not only on the formal institutional labels but is also present in handwritten notes on the labels and on the specimen sheet itself. The research value of these specimens is maximised when all data present on a specimen and derived digital image are transcribed verbatim, those data are then enriched and/or interpreted and recorded in the collection management system, so that specimen data becomes searchable and available to other researchers. A first step toward reducing the manual labour‐intensive task of initial verbatim data transcription is building a means for artificial intelligence to identify areas where these data are present on the SSDI.

Much of the earlier literature addressing this task concentrates on extracting data from labels via optical character recognition (OCR). Some applied OCR software to the whole SSDI, (e.g. Drinkwater et al., 2014; Haston et al., 2012; Tulig et al., 2012). Other studies identified the label first and then applied OCR; in these cases, selecting or ‘marking up’ the label area was either (a) manual, (e.g. Alzuru et al., 2016; Anglin et al., 2013; Barber et al., 2013; Dillen et al., 2019; Haston et al., 2015); (b) vaguely described, (e.g. Heidorn & Wei, 2008; Takano et al., 2019, 2020); or (c) proposed as future work (i.e. not actually implemented) (e.g. Haston et al., 2015; Kirchhoff et al., 2018; Moen et al., 2010). Some investigations (e.g. Alzuru et al., 2016; Haston et al., 2015; Owen et al., 2019) demonstrated that applying OCR tools to the label‐only images was more effective, faster and more accurate, than applying OCR tools to the whole SSDI. Owen et al. (2019) took this a step further and found that running OCR over individual text lines cropped from a label image was faster than processing the whole label. These findings reinforce the value of pursuing the current research, for having a semi‐automated tool which identifies components of an SSDI, which can then be cropped out and further analysed/transcribed, holds potential for downstream elements in the SSDI data collection to be more efficient. Automated identification of components of specimen images lends itself to the application of computer vision (CV) models.

In recent years computer vision models have become more sophisticated (for literature reviews see Hussein et al., 2022, Rocchetti et al., 2021, Wäldchen & Mäder, 2018). While some studies have applied CV methods to the analysis of the plant material, here the application of that technology to identify label and handwritten data is of most interest. Relevant forms of CV include object detection, classification, and semantic segmentation. Semantic segmentation is at the pixel level (Nieva de la Hidalga et al., 2022; Triki et al., 2022; White et al., 2020), whereas object detection methodology uses bounding boxes. And while there is ‘some overlap between semantic segmentation and object detection’ (Walton, Livermore, & Bánki, 2020; Walton, Livermore, Dillen, et al., 2020, p. 7), the latter can be used ‘to identify and segment the different objects that are commonly found on herbarium sheets’ (ibid., p. 7). One such tool is YOLO (*Y*ou *O*nly *L*ook *O*nce, Redmon et al., 2016). The third version, YOLOv3, was applied to SSDIs by Triki et al. (2020, 2022); in that study, 4000 SSDIs from the Freidrich Schiller University Jena herbarium Germany (JE) were manually marked‐up and used to train a model to identify specific plant traits and organs. Nieva de la Hidalga et al. (2022) also used YOLOv3 when cross‐validating The Natural History Museum London semantic segmentation network (NHM‐SSN) on a collection of microscope slides.

This paper describes efforts to identify all components of a digital image of an herbarium specimen sheet by training a YOLOv5 object detection model on a subset of MELU SSDIs. As the building of this capacity is itself resource‐intensive with respect to time, expertise and computational infrastructure—with smaller and medium‐sized collections regularly resource constrained—the key aim was to derive and share practical guidelines to enable other herbaria to integrate such a model in their digitisation workflow. As such, the specific research questions were:

1. Can a model be built to separately identify labels, handwriting and other original information, taxon annotation labels and other components of a specimen sheet digital image?
2. How many images must be annotated to train an effective model?
3. What is required to enable cross‐herbarium application of the model, that is, how many new annotated images are needed to retrain a model for a new feature or collection?

## 2 METHODOLOGY

To answer the first research question, an object detection model was built. The second research question was interrogated by testing model parameters. The third research question involved testing how many additional marked‐up images were needed to retrain the model to accurately identify a new feature.

### 2.1 Choosing YOLOv5

It is usually less labour‐intensive to mark up training data for an object detection model than for a semantic segmentation model. With this in mind, taking into account the heterogeneity of the MELU SSDIs and that a substantial number of images would be required for any model, and considering the methods observed in the reviewed literature, an object detection model using YOLOv5 (https://github.com/ultralytics/yolov5) was chosen for this investigation (described more below). While a comparative study against other methods and models is a promising research area, the focus of this investigation was to comprehensively investigate and quantify what accuracy could be achieved using this specific model type.

YOLO works through a single neural network base to predict bounding boxes around objects and class probabilities for those boxes (Redmon et al., 2016). The model uses a series of convolutional layers to infer features from the whole image and reduce the size of the spatial dimensions. Detections for the bounding boxes and class probabilities are made on coarse spatial cells resulting from the convolutions and predictions of the same object in multiple cells are corrected using non‐maximal suppression. Enhancements were made to the model in the release of YOLO9000 (Redmon & Farhadi, 2017) and YOLOv3 (Redmon & Farhadi, 2018). A Python implementation of this model using PyTorch was released in 2020, named YOLOv5 (Jocher, 2020). This implementation of YOLO was used for this project for its convenience and flexibility. All YOLO training and validation were run on the University of Melbourne's high‐performance computing infrastructure using four Intel Xeon E5‐2650 CPUs and a single NVIDIA Tesla P100 GPU.

### 2.2 Phase 1. MELU‐trained model

SSDIs from MELU were annotated. A subset of these images was used to train an object detection model, and the remaining SSDIs validated the accuracy of the trained model. Training and validation were then undertaken on various‐sized training datasets, also varying modelling parameters. The output is the MELU‐trained ‘sheet‐component model’ and recommendations for how many annotated images are required to train an effective model.

#### 2.2.1 Annotating MELU images

Both medium‐ and high‐resolution MELU SSDIs were downloaded from the publicly accessible collection portal (https://online.herbarium.unimelb.edu.au/). In the machine learning context, to ‘annotate’ a SSDI is to mark up the image to identify the areas of interest. Contrary then to how the word ‘annotation’ is used in the herbarium curation field, here is it used to refer to the information from the marking up exercise.

**FIGURE 2:**
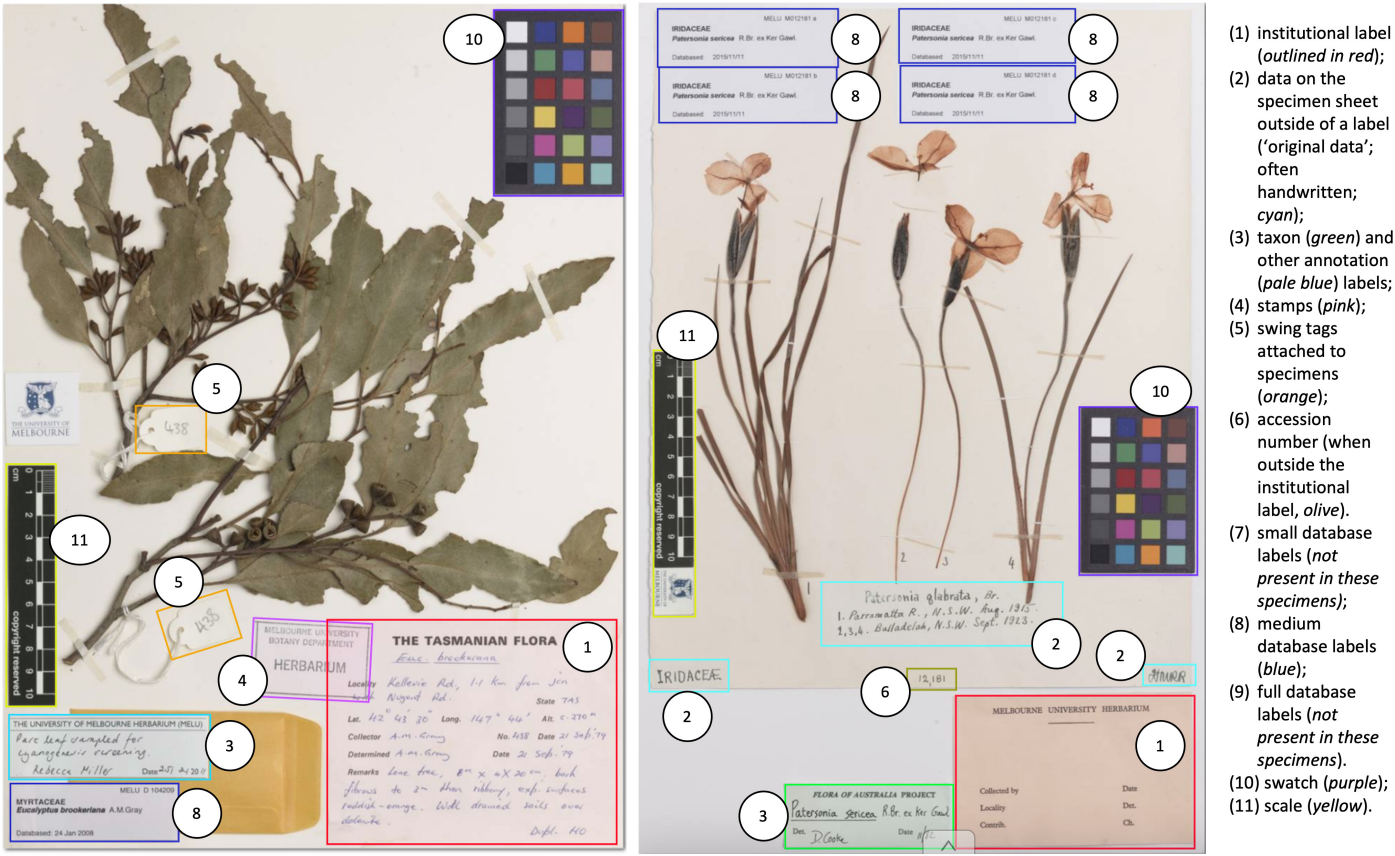
Annotated images from MELU; (left) MELUD104209 (https://online.herbarium.unimelb.edu.au/collectionobject/MELUD104209a), (right) MELUM012181a (https://online.herbarium.unimelb.edu.au/collectionobject/MELUM012181a).

The MELU curator, together with the analytic team, determined SSDI components, or areas of interest. The guiding principles of this part of the study were to maximise the potential value from the annotation exercise, and, therefore, all components on the SSDIs except for the biological specimen were annotated. In this way, this data could be made available for future (as yet unforeseen) summaries and investigations, and the object detection models for this investigation could be consolidated if the analysis suggested this was required. Figure 2 shows two examples of annotations on MELU SSDIs: (1) institutional label; (2) data on the specimen sheet outside of a label (‘original data’, often handwritten); (3) taxon and other annotation labels; (4) stamps; (5) swing tags attached to specimens; (6) accession number (when outside the institutional label). Also of interest were labels produced as part of the MELU digitisation process: (7) small database labels; (8) medium database labels; (9) full database labels. Further, artefacts from the imaging process that do not remain with the specimen sheet: (10) swatch; (11) scale. When a marked‐up box is given one of the above names, they are usually called labels; however, given the context, they will be referred to as component categories here. Often there was more than one component per sheet (especially for taxon annotation labels), and the colour swatch was sometimes broken into two parts. While the examples in Figure 2 show horizontally oriented institutional labels, MELU also has examples of vertical orientation.

The annotation work was undertaken in an online open‐source tool CVAT (*C*omputer *V*ision *A*nnotation *T*ool; https://cvat.org/). CVAT permits storing these annotations in a number of formats, including a.JSON format (.COCO type) and a zip file of .TXT files, one for each image (.YOLO format). Of the 13,095 available MELU SSDIs, 4371 were manually annotated in CVAT. Almost all annotating was undertaken by one person, who also checked the remaining annotations made by others. This approach avoided inter‐annotator variability. In this paper, the phrase ‘image‐annotations’ is used to refer to the set of annotations for a set of SSDIs, not the actual count of those annotations, that is, a total of 4371 image‐annotations are available for use.

The annotation data were used to generate collection summaries to identify how common each component was on MELU SSDIs. These data were also used to locate the centre point of each of the SSDI components on the specimen sheets, using two‐dimensional kernel density estimations (KDE) to create locative ‘heat maps’.

#### 2.2.2 YOLOv5 model training and testing

The *MELU‐trained sheet‐component* YOLOv5 model was trained using the medium‐resolution images, a validation dataset of 1000 image‐annotations randomly selected from the 4371 available, with the remaining 3371 image‐annotations forming the training dataset. The model parameters were selected after testing: the ‘large’ pre‐trained YOLOv5 model type, on 640 pixels, run for 200 epochs. As detailed further below, different and smaller validation and training datasets were also created using this original whole set of image‐annotations, to test YOLOv5 model parameters with the specific aim of determining what size training dataset size balanced model accuracy and runtime trade‐offs. Then, to identify optimum model parameters, some of these models were also trained with different YOLOv5 model parameters: epoch count, either ‘large’ or ‘medium’ model size type, either 640 or 1280 pixel resolution.

There is potential for the heterogeneity of the layouts and components in the 4371 MELU SSDIs to influence the model training results, such that the derived model may work well for SSDIs with a frequently encountered layout and poorly for SSDIs that were infrequently encountered in the training dataset. To separate the potential bias from individual image‐annotations from the actual impact driven by training dataset size 10 new training datasets of each sample size were randomly drawn (with replacement) from the full training dataset. Similarly, the makeup of the validation dataset may influence model outcomes, therefore four new validation datasets and four new sets of accompanying training datasets were also created.

In total, 282 training‐validation dataset combinations (detailed in Table A1) provided indications for the impact of significant SSDI heterogeneity, and guidance for determining how many images must be annotated to train an effective model.

#### 2.2.3 Assessing trained models

Measures used to evaluate the accuracy of the trained models were: (i) precision; (ii) recall; (iii) F1; (iv) mAP0.5; (v) mAP0.5–0.95; and (vi) confusion matrix. These measures are well described elsewhere (e.g. Redmon et al., 2016), but as mAP0.5–0.95 is used as the key measure in this work a brief description is worthwhile. Mean average precision (mAP) is effectively a combination of the precision and recall measures, it is between 0 and 1, and the higher the value the better the model. It effectively measures the overlap between the actual and predicted object boundaries (i.e. the ‘intersection over union’ (IoU)). For example, mAP0.5 is the mAP where the boundaries overlap by at least 50%. Then, mAP0.5–0.95 is the average mAP for IoU between 50% and 95% overlaps in 5% steps. These measures were visualised using the web‐based tool Weights and Biases (https://wandb.ai/). Each component category (e.g. ‘institutional label’, ‘swatch’) is assessed separately for these measures, and the overall model measures are an arithmetic average across the component categories. When assessing a trained model, YOLOv5 assigns the ‘best’ epoch for a model is that with the highest value for (10% mAP0.5 + 90% mAP0.5–0.95).

### 2.3 Phase 2. Applying the sheet‐component model to unseen images

**FIGURE 3:**
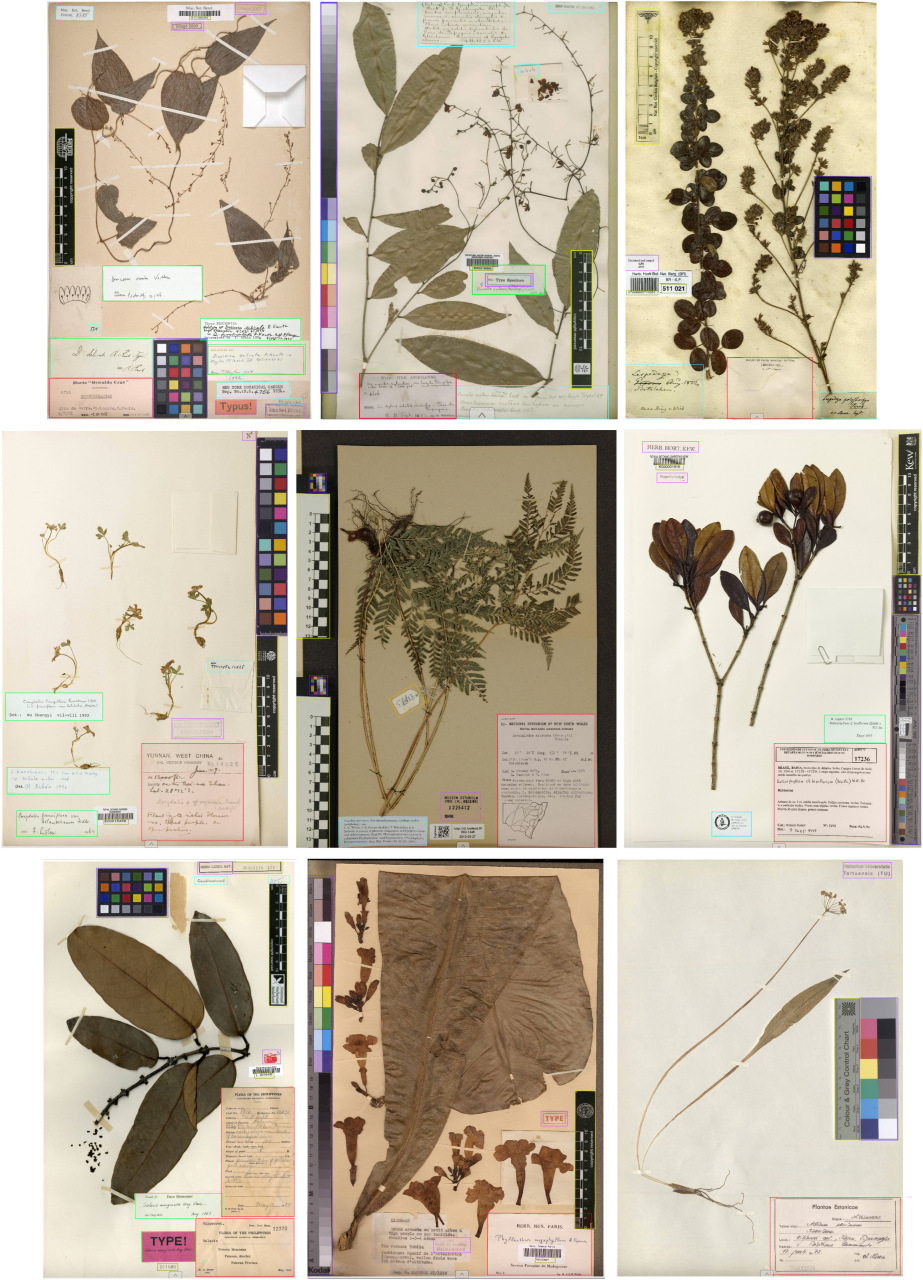
Example benchmark images (from Dillen et al., 2019), annotated for MELU testing (see Figure 2 for colouring): top row: B, B100000389 (http://herbarium.bgbm.org/object/B100000389) (left), BM, BM000798595 (http://data.nhm.ac.uk/object/a4887457‐02bc‐4099‐b1e8‐9804c837d1a0) (middle), BR, BR0000005110216 (http://www.botanicalcollections.be/specimen/BR0000005110216) (right) middle row: E, E00015458 (http://data.rbge.org.uk/herb/E00015458) (left), H, EIG.1345 (http://id.luomus.fi/EIG.1345) (middle), K, K000001916 (http://specimens.kew.org/herbarium/K000001916) (right) bottom row: L, L0015432 (http://data.biodiversitydata.nl/naturalis/specimen/L%20%200015432) (left), P, P00093937 (http://coldb.mnhn.fr/catalognumber/mnhn/p/p00093937) (middle), TU, TU253504 (https://plutof.ut.ee/#/specimen/view/147673) (right).

The purpose of Phase 2 is to go some way towards answering the third research question. To quantify the MELU‐trained model's transferability, a subset of SSDIs from the benchmark dataset published by Dillen et al. (2019) was used as a test dataset (referred to as the ‘benchmark dataset’ in this paper). This research made available 1800 images, 200 images each from nine herbaria, each represented by their official herbarium acronym: B (Botanical Garden and Botanical Museum, Berlin, Germany), BM (Natural History Museum, London, England), BR (Meise Botanic Garden, Meise, Belgium), E (Royal Botanic Garden, Edinburgh, Scotland), H (Finnish Museum of Natural History, LUOMUS, University of Helsinki, Helsinki, Finland), K (Royal Botanic Gardens, Kew, England), L (Naturalis Biodiversity Centre, Leiden, Netherlands) and P (National Museum of Natural History, Paris, France). The first 51 images of each herbaria were annotated. When annotating the new SSDIs in CVAT some compromises were required, as it was undesirable to change or add to the categories used for training the MELU model. For example, the combined swatch‐scale component in SSDIs in the benchmark dataset was categorised as ‘swatch’; barcodes were ignored where possible, but the numbers under or near them were categorised as ‘accession number’. It was not expected that the MELU‐trained model would cope well with these components as it was not trained on them. Examples of image‐annotations are in Figure 3. The annotation data was also used to locate the centre points of SSDI components, for comparison to MELU SSDI heat maps.

The MELU‐trained model was initially tested using annotations from each of the nine herbaria separately and then tested against the combined set of benchmark dataset image‐annotations. The heterogeneity of the SSDI components and layouts from each herbarium means an ‘overall’ result was less useful than individual results. Precision, recall, mAP0.5 and mAP0.5–0.95 along with the confusion matrix were used for the assessment of model accuracy.

### 2.4 Phase 3. Retraining for new/additional images

The purpose of Phase 3 is to answer the third research question, specifically: how few annotations are needed to retrain the MELU‐trained model for a feature that the model was not trained on.

#### 2.4.1 Adding new annotations to the MELU training dataset

From the 51 image‐annotations of SSDIs from each herbarium in the benchmark dataset, a validation dataset of 20 image‐annotations were randomly chosen and the remaining were assigned to the 30 image‐annotation training dataset (one image‐annotation was ignored). Smaller training datasets, of 20 and 10 image‐annotations, were then also created by progressively removing image‐annotations from the 30 image‐annotation training dataset. In this set of tests, the MELU‐trained model was validated against the 20 image‐annotation validation dataset to create baseline measures for subsequent comparisons. A new model was then trained on a dataset that combined both the full MELU training dataset and the 30 image‐annotation training dataset, beginning with weights from MELU‐trained sheet‐component model and run for 50 epochs. Additional new models were subsequently trained using the full MELU training dataset plus each of the 10 and 20 image‐annotation training datasets, to gauge how few new annotations were required to produce a reasonable model.

#### 2.4.2 Only using new annotations

The purpose of this group of tests was to determine whether retraining the MELU‐trained model only on the additional image‐annotations, without including the full MELU training dataset, could be as effective for developing an accurate model. The expectation was that these tests would be faster and, therefore, more practical for other herbaria if the results were comparable.

For efficiency, this investigation combined the data from the herbaria in the benchmark dataset. First, a new model was trained with 10 image‐annotations per benchmark herbarium (i.e. 90 in total) that were added to the full MELU training dataset, with the validation dataset built from the remaining 40 image‐annotations from each benchmark herbarium (i.e. 360 image‐annotations). Next, a new model was trained using *only* these 90 training image‐annotations and the same validation dataset. Both models began with weights from the MELU‐trained sheet‐component model; the full MELU training dataset plus benchmark training dataset was run for 40 epochs and the benchmark‐only dataset was run for 30 epochs.

The individual SSDIs may bias model results using such small training and validation datasets. To mitigate this, four additional 10‐, 20‐ and 30‐ image‐annotation training datasets were generated including SSDIs from each herbarium in the benchmark dataset. Four mutually exclusive training datasets of 10‐, 20‐ or 30‐image‐annotations were constructed (producing datasets of 90, 180, 270 image‐annotations in total, respectively) and the remaining 40‐, 30‐, 20‐ image‐annotation were assigned to the validation dataset. A total of five datasets were therefore available for model testing. The average model outputs from these five datasets, derived using image‐annotations from the benchmark herbaria only, were then compared to the output from the model from the full MELU training dataset plus the benchmark training dataset.

## 3 RESULTS

### 3.1 Phase 1: MELU SSDI annotations

In total 4371 MELU images were annotated resulting in 24,666 individual annotations. Annotation counts of the MELU SSDI components are presented in Table 1 by component type. Reading the information for ‘annotations taxon’ as an example: 3126 SSDIs do not have this component (i.e. in the ‘0’ column); 987 of these have only one taxon annotation label on the sheet, 213 SSDIs have two, 1 SSDI has five and, therefore, the total number of annotations for this component is 1554; and this component is present on 28% of SSDIs (987 + 213 + 40 + 4 + 1 = 1245, and 1245/4371 = 28%).

**TABLE 1:** Counts of annotations of MELU SSDIs.

Component	Count of each component on the specimen sheet												Total annotation count	Count of images with component	% of Images with component
	0	1	2	3	4	5	6	7	8	9	10	12			
Photographing artefacts															
Scale	2	4369											4369	4369	100
Swatch (pieces)	1	4326	43	1									4415	4370	100
Of most interest for data gathering															
Institutional label	1072	2967	323	9									3640	3299	75
Annotation—taxon	3126	987	213	40	4	1							1554	1245	28
Annotation—other	4288	82	1										84	83	2
Stamp	3170	1149	51		1								1255	1201	27
Swing tag	3604	720	40	6	1								822	767	18
Handwriting	2960	670	279	324	96	23	8	4	3	1	1	1	2830	1410	32
Number	3735	560	61	10	5								732	636	15
Digitisation Labels															
Full database label	3347	1023	1										1025	1024	23
Database label	835	3306	147	59	14	9	1						3884	3536	81
Small database label	4348	14	2	1	1	1	2	2					56	23	1
													24,666		

When the whole set of annotations was split between the training and validation datasets, the proportions across each component were checked, to ensure the two datasets were not biased. As demonstrated in Table 2, the proportions (the ‘% of annotations’ columns) are similar. As is the average count of annotations per SSDI.

**TABLE 2:** Splits of annotations of MELU SSDIs, in total and across training and validation datasets.

	MELU‐trained object detection model				All available annotations	
	Training dataset		Validation dataset			
Total count of images	3371		1000		4371	
**Component**	**Annotation count**	**% of annotations**	**Annotation count**	**% of annotations**	**Annotation count**	**% of annotations**
Photographing artefacts						
Scale	3369	18	1000	18	4369	18
Swatch (pieces)	3409	18	1006	18	4415	18
Of most interest for data gathering						
Institutional label	2816	15	824	14	3640	15
Annotation—taxon	1254	7	384	7	1638	7
Annotation—other						
Stamp	957	5	298	5	1255	5
Swing tag	636	3	186	3	822	3
Handwriting	2148	11	682	12	2830	11
Number	551	3	181	3	732	3
Digitisation labels						
Full database label	796	4	229	4	1025	4
Database label	2991	16	893	16	3884	16
Small database label	29	0	27	0	56	0
	18,956	100	5710	100	24,666	100
Avg. count of annotations per SSDI	5.6		5.7		5.6	

**FIGURE 4:**
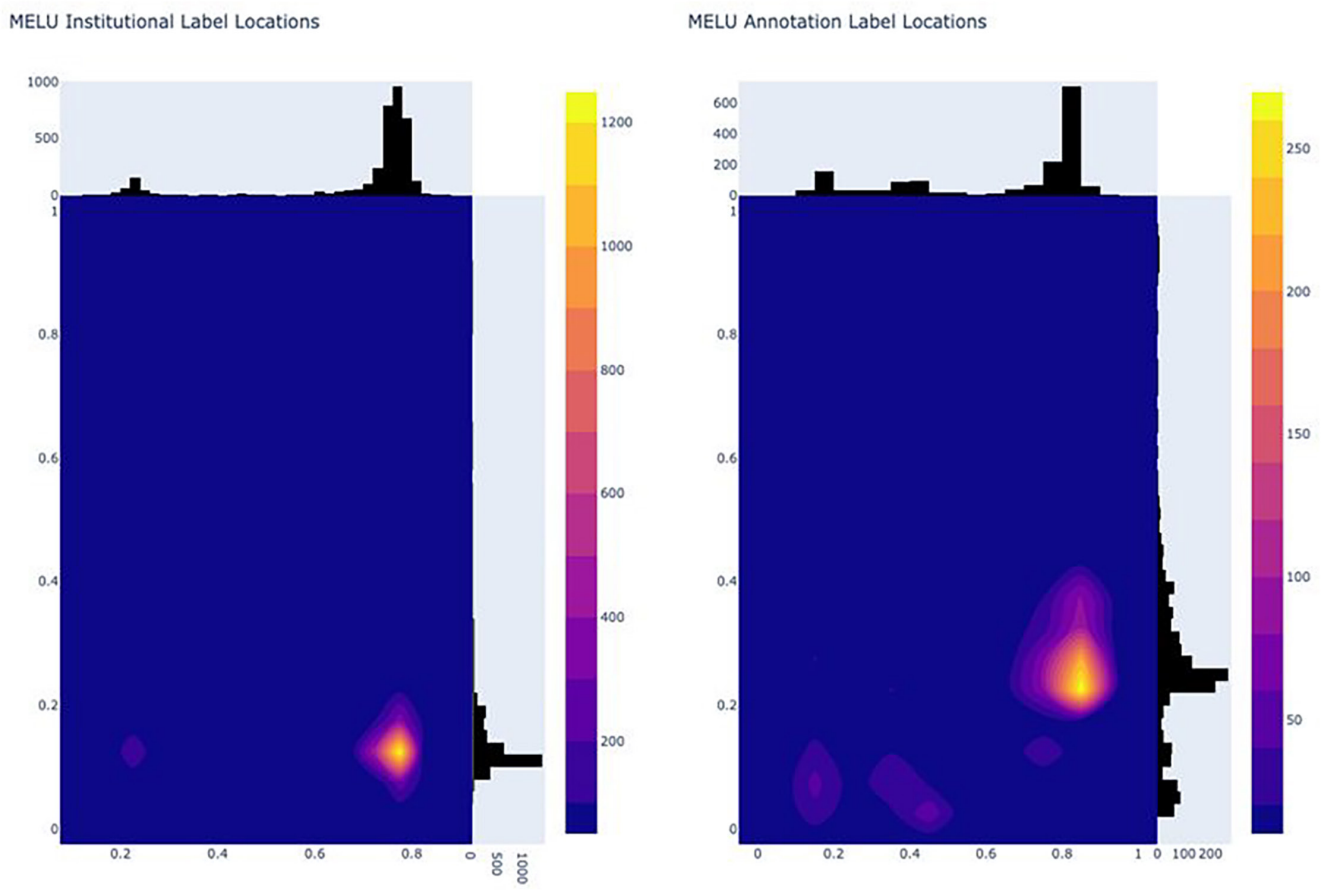
‘Heat maps’ of the centre‐point of (left) institutional label and (right) annotation labels for 4371 MELU annotated images SSDIs.

The ‘heat maps’ for the centre of the institutional (left) and annotation (right) labels are presented in Figure 4.

### 3.2 Phase 1: MELU‐trained sheet‐component object detection model

The final model was run using YOLOv5 parameters: large model, 640 pixels, 200 epochs (self‐curtailed at 159 epochs). After completion of the initial Phase 1 tests, manual review of the annotations led to a small number of revisions: some box boundaries were tightened, and some swing tags were given diagonally aligned, more fitting, boxes which were used for revised Phase 1 test completion. The small differences in the model measures (Table 3) between the *initial* and *revised* model confirmed that the comparisons detailed in this section—all from the *initial* set of image‐annotations—remain valid.

**TABLE 3:** Comparison of model assessment measures for initial and revised MELU‐trained sheet‐component object detection model.

MELU‐trained model	Precision	Recall	mAP0.5	mAP0.5–0.95
Initial	0.976	0.974	0.980	0.837
Revised (minor annotation revisions)	0.983	0.969	0.979	0.847

**FIGURE 5:**
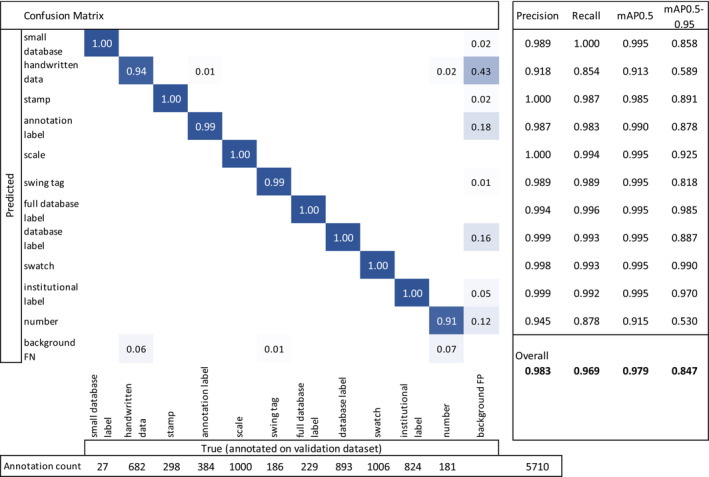
Confusion matrix and model assessment measures for best epoch of revised run.

The confusion matrix for the ‘best’ epoch of the *revised* model (Figure 5) indicates how each of the sheet‐component categories is being predicted. The numbers on the diagonal are the proportion of predictions that are correct (true positive). Here, the model consistently correctly predicts ‘institutional label’, ‘full database label’, ‘database label’, ‘stamp’, ‘scale’ and ‘swatch’ annotations (i.e. 1.00). ‘Accession number’ and ‘handwritten data’ appear difficult to consistently predict with confidence (at 0.91 and 0.94, respectively). The right column in the matrix shows each component category as a percentage of all background false positives, which are areas on the SSDI erroneously predicted by the model to be a component. Background false positives are highest for ‘handwritten data’ (0.43) and low for ‘institutional labels' (0.05), and there are none for ‘scale’ and ‘full database label’. Model accuracy results (right table in the Figure), particularly mAP0.5–0.95, indicate that the prediction of ‘institutional label’ (at 0.970) is much stronger than that of ‘annotation label’ (0.878) and both are more accurately predicted than ‘handwritten data’ (0.589) and ‘number’ (0.530) components.

### 3.3 Phase 1: Testing trained models

Early in the testing regime, it was found that the ‘large’ YOLOv5 model type produced better models than the ‘medium’ model type with minimal time trade‐off. It was also found that running on 1280 pixels took more than three times longer than running on 640 (e.g. while specific to the infrastructure used in this study, a ‘large’ model trained on the 1500 sample for 200 epochs on 640 pixels took 8.6 h, and 29.7 h when run on 1280 pixels). Given the capacity to run the 640 for more epochs, it was felt that the results were not sufficiently different at 1280 for the time spent. Therefore, all further tests were run using ‘large’ model type and 640 pixels.

**FIGURE 6:**
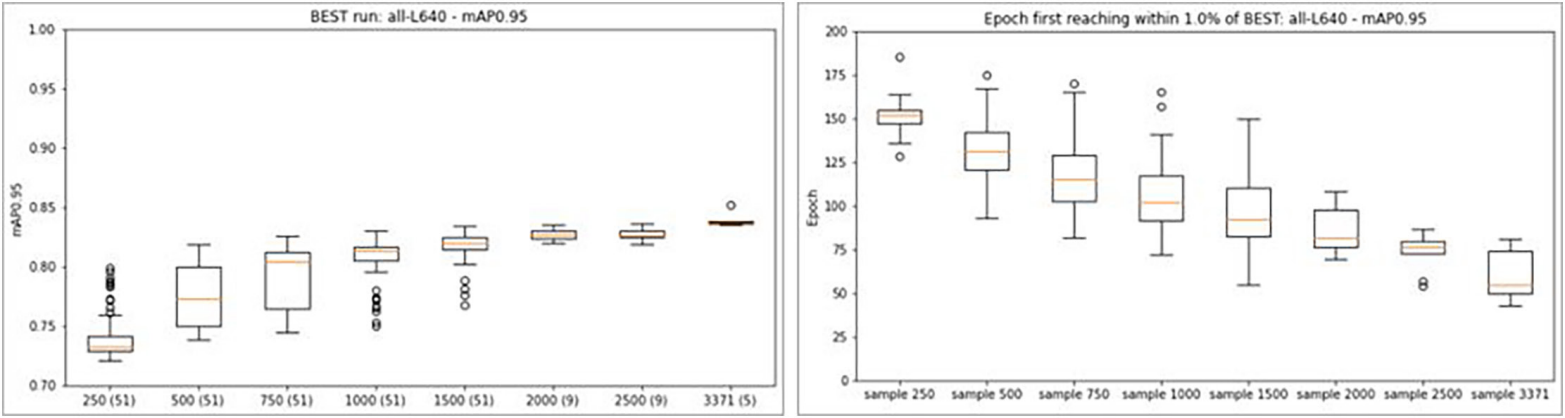
(Initial) models run on large/640/200; (number in brackets is count of runs for that size training dataset); (left) mAP0.5–0.95 measure; (right) epoch when first reach within 1% of the best mAP0.5–0.95.

Trials show that mAP0.5–0.95 is the highest for models trained on larger validation datasets, as indicated by the ‘best’ epoch for a model (Figure 6, left). The median mAP0.5–0.95 (orange bar in graph) increases and variability (indicated by the size of rectangle and whiskers in graph) decreases as training dataset size increases. Taking the models run on training datasets of size 750 SSDI image‐annotations as an example of how to read the graph: 51 models were tested with different image‐annotations included in the training and validation datasets; the median mAP0.5–0.95 for these was 0.80 (95% confidence intervals 0.75–0.83). Further, the larger the training dataset, the quicker the model attains stability, as indicated by the epoch at which the model was first within 1% of the eventual ‘best’ mAP0.5–0.95 (Figure 6, right). Following the same example: the median number of epochs for the 51 runs to reach within stability was approximately 120 (95% confidence intervals 80–170).

**FIGURE 7:**
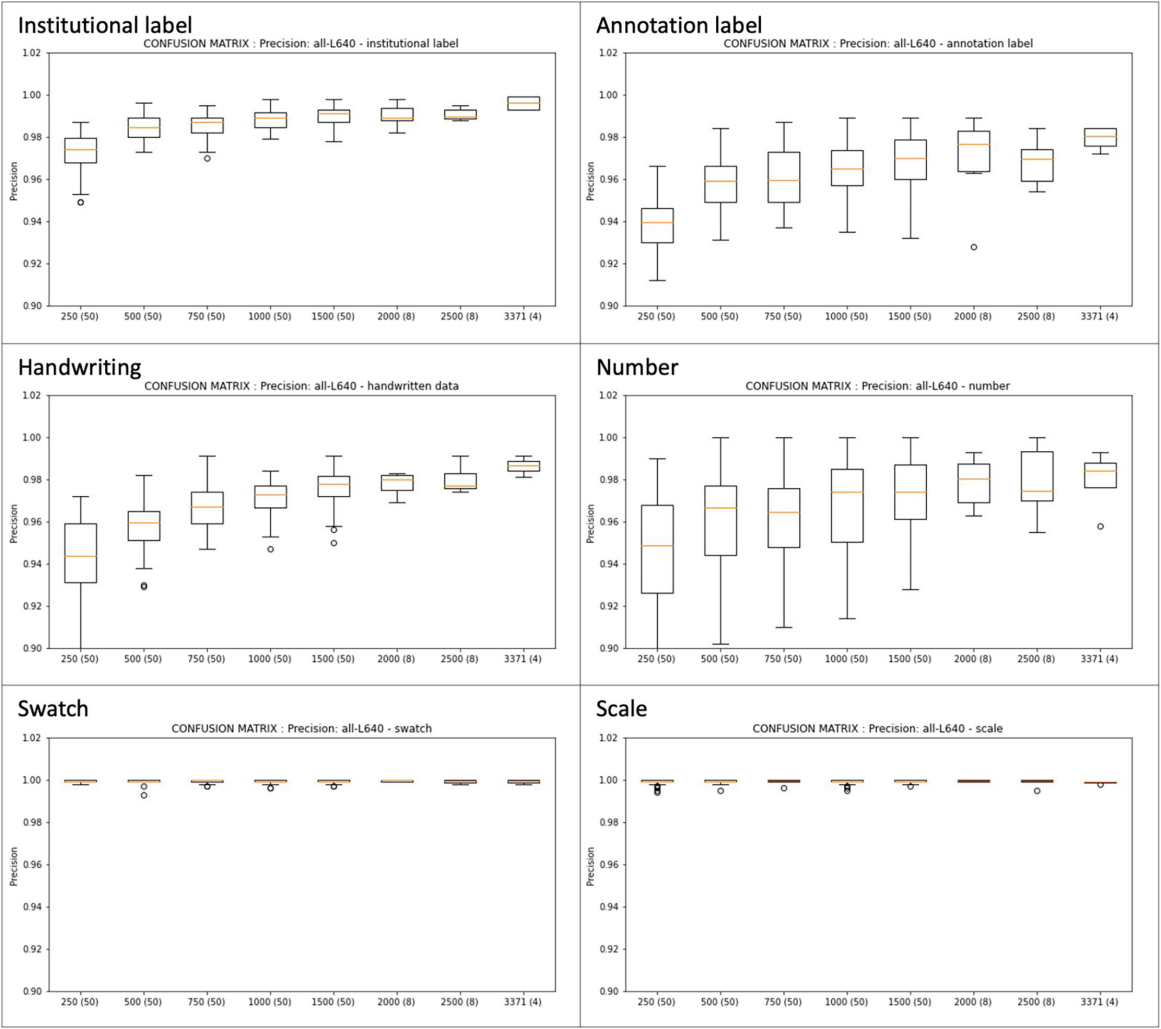
Precision measures (original tests, excl. full revised model) by key component; at ‘best’ epoch for each run (note constrained y axis).

The variable predictability of each component type influences overall model outcomes. Precision for each component, assessed at the ‘best’ epoch of each model (Figure 7) across the runs for each sample training dataset size (*x* axis), behaved similarly to the mAP0.5–0.95 parameter of the overall *revised* model; that is, median increases and variability decreases as training dataset size increased. Additionally, components with good overall predictability in the full model (per mAP0.5–0.95 in Figure 5; for example, scale, institutional label) showed less variability across all training dataset sizes than the poorly predicted components (e.g. number).

### 3.4 Phase 2: Benchmark dataset annotations

Table 4 counts the image‐annotations from the 51 annotated SSDIs for each benchmark dataset herbarium used in this phase, by component type.

**TABLE 4:** Counts of annotations for each herbarium in benchmark Dillen et al. (2019) study.

Benchmark annotations	B	BM	BR	E	H	K	L	P	TU	ALL
Original data	19	104	51	36	36	41	41	31	5	364
Stamp	126	43	63	56	52	87	103	26	49	605
Annotation label	46	73	13	59	40	63	79	32	18	423
Scale	51	39	51	52	102	51	37	20	51	454
Swing tag	2	4	1	5	2	9	15	2	0	40
Swatch	19	51	51	51	102	59	54	20	36	443
Institutional label	52	47	92	50	53	57	51	75	52	529
Number	88	74	132	66	51	66	162	90	9	738
Overall	403	435	454	375	438	433	542	296	220	3596
Overall ex ‘swing tag’	401	431	453	370	436	424	527	294	220	3556

**FIGURE 8:**
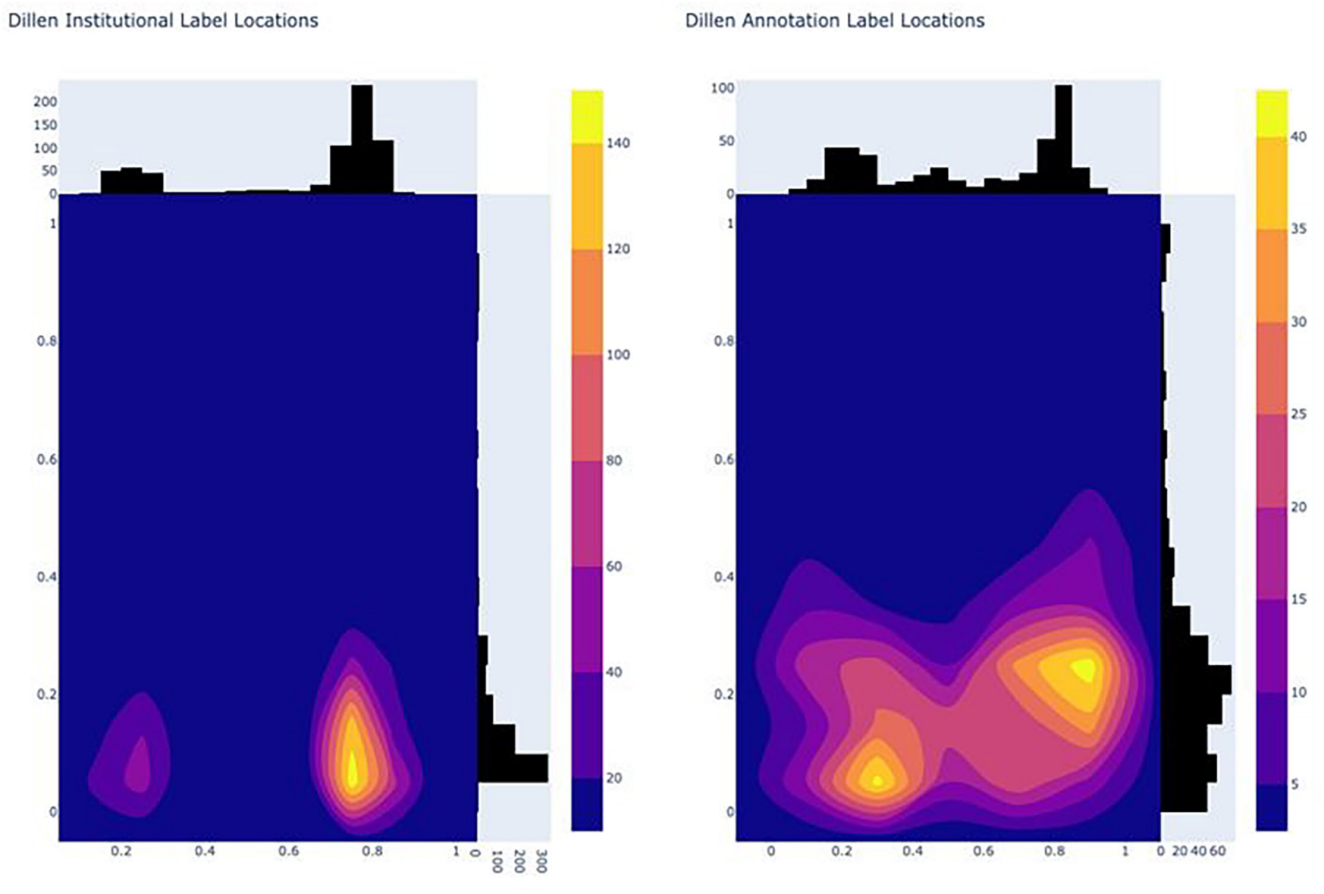
’ Heat maps' of the centre‐point of (left) institutional label and (right) annotation labels for annotated Benchmark SSDIs.

The ‘heat map’ of centre points for institutional (left) and annotation (right) labels for the SSDIs in the benchmark dataset is shown in Figure 8 and enables comparison to placement in the MELU SSDIs (Figure 4).

### 3.5 Phase 2: Applying the MELU model to unseen SSDIs

**FIGURE 9:**
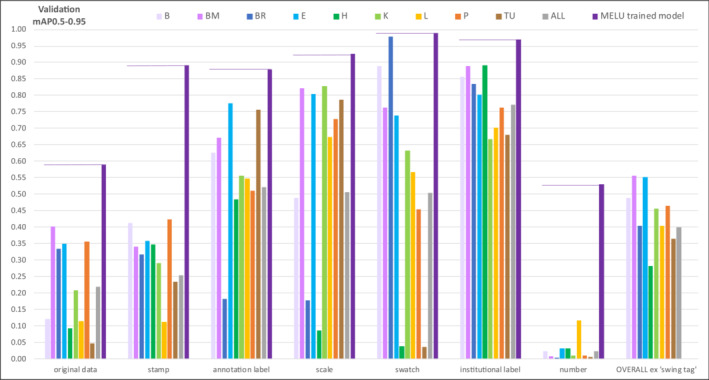
Validating against benchmark SSDIs, comparison of mAP0.5–0.95.

Validating the *revised* MELU‐trained object detection model against each of the benchmark datasets produced different results by herbarium (Figure 9). The ‘institutional label’ component was reasonably well identified across all (mAP0.5–0.95 of 0.68–0.89). However other components—for example, ‘scale’ for Meise Botanic Garden (BR; mAP0.5–0.95 of 0.18) and University of Helsinki (H; mAP0.5–0.95 of 0.09), and ‘swatch’ for Helsinki (mAP0.5–0.95 of 0.04) and University of Tartu (TU; mAP0.5–0.95 of 0.04)—were all but ignored by the model. Additionally, for each SSDI component, prediction precision varies among herbaria. For example, the ‘scale’ component ranges from a high mAP0.5–0.95 value of 0.83 for Kew (K) SSDIs to a low mAP0.5–0.95 value of 0.09 Helsinki SSDIs. Note that in these analyses, ‘All’ is not an average across the nine herbaria but was run separately with all available SSDIs combined.

### 3.6 Phase 3: Adding new annotations to the MELU training dataset

**FIGURE 10:**
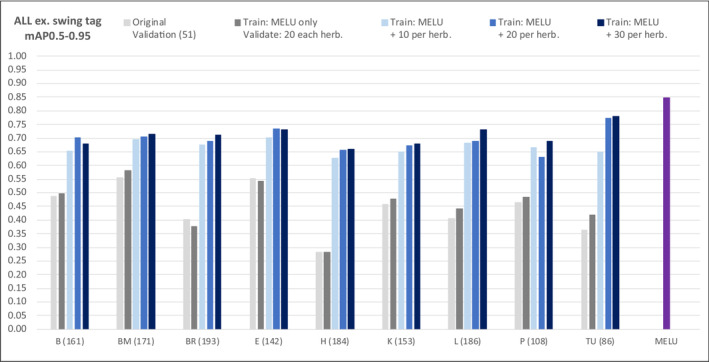
Retraining tests, across all components, by herbarium (values in brackets on x axis are count of image‐annotations in validation dataset).

**FIGURE 11:**
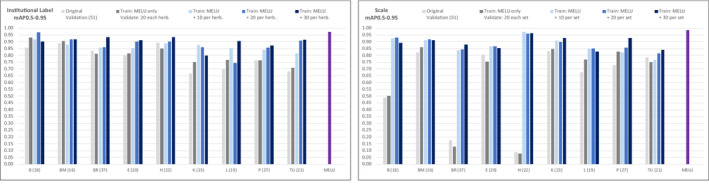
Retraining tests by herbarium, ‘institutional label’ (left) and ‘scale’ (right).

Greater precision for the prediction of SSDI component types was achieved when a new model was trained on a dataset that combined both the fully *revised* MELU training dataset *and* image‐annotations from each herbarium in the benchmark dataset. Figure 10 graphs mAP0.5–0.95 per herbarium for all components together for validation and each additional training test with 10, 20 and 30 additional image‐annotations per herbarium. The *grey* bars are validation runs and are included for comparison: *pale grey* as per Figure 9, and the *dark grey* bar is the validation baseline for this set of tests. The *blue* bars are new models trained when new benchmark image‐annotations were added to the full MELU training dataset: adding 10 (*pale*), 20 (*medium*), or 30 (*navy*). The *purple* bar to the right is from the original MELU‐trained model, for comparison. The mAP0.5–0.95 values incrementally increase with the incorporation of the successively larger training datasets. For example, the MELU model applied to 20 validation image‐annotations from Helsinki (H) without any retraining predicts (across all components) with mAP0.5–0.95 of 0.28, but when as few as 10 image‐annotations are added and the model is retrained with these alongside the full MELU training dataset, the mAP0.5–0.95 increases to 0.63. For more detail on key components on interest, Figure 11 illustrates the change for ‘institutional label’ (left), and ‘scale’ (right). For the latter, retraining with 10 image‐annotations for Helsinki has increased mAP0.5–0.95 from 0.08 to 0.97.

Adding as few as 10 new image‐annotations per herbaria (Figure 10) resulted in mAP0.5–0.95 increasing by an average of 0.21 across herbaria (0.11–0.35; difference between *dark grey* and *pale blue* bars). Adding 20 new image‐annotations benefited Estonia (TU) more than other herbaria; though it was unexpected that the 20‐set was marginally less predictive than the 10‐set for Paris (P).

Focussing now on ‘institutional label’ (Figure 11, left), except for Berlin (B), adding 30 new image‐annotations again improved the predictions. Adding 30 does not produce better results than adding 20 (*medium blue*) for Berlin and Kew (K). Additionally, adding 10 (*pale blue*) image‐annotations appears to give a more predictive model than adding 20 image‐annotations for herbaria Kew and Leiden (L); and adding 20 better than 30 for Berlin and Kew.

### 3.7 Phase 3: Only using new annotations

Table 5 details mAP0.5–0.95 for various models tested in this phase, by component, for each of the 10, 20, or 30 benchmark image‐annotation (per herbaria) dataset tests.

**TABLE 5:** Comparing running with MELU training dataset, against only training with new benchmark image‐annotations (all sets combined).

ALL herbaria—by component (ex. swing tag)	Train: MELU +10 per herb.	Train: ONLY 10 per herb. Avg runs 1–5	Advantage of training WITH MELU	Train: MELU +20 per herb.	Train: ONLY 20 per herb.	Advantage of training WITH MELU	Train: MELU +30 per herb.	Train: ONLY 30 per herb.	Advantage of training WITH MELU
	(a)	(b)	(a)–(b)	(c)	(d)	(c)–(d)	(e)	(f)	(e)–(f)
Original data	0.32	0.33	−0.01	0.36	0.34	0.02	0.34	0.35	−0.01
Stamp	0.62	0.59	0.03	0.64	0.65	−0.01	0.68	0.67	0.01
Annotation label	0.72	0.70	0.02	0.76	0.75	0.01	0.73	0.74	−0.01
Scale	0.90	0.87	0.03	0.89	0.89	0.00	0.92	0.92	0.00
Swatch	0.91	0.89	0.02	0.91	0.90	0.01	0.92	0.92	−0.01
Institutional label	0.87	0.87	0.00	0.90	0.90	0.00	0.91	0.91	0.00
Number	0.49	0.44	0.05	0.50	0.51	−0.01	0.52	0.53	−0.01
Overall	0.69	0.67	0.02	0.71	0.71	0.00	0.72	0.72	0.00

The first column in each block is the mAP0.5–0.95 for model trained with new annotations *added* to the full MELU training dataset. These are then compared to the new models trained *only* on the benchmark image‐annotations. The third column is the difference. As an example: the model trained using *only* the 10 new image‐annotations per herbarium appears to have the slightly less predictive power for ‘scale’ (mAP0.5–0.95 of 0.87) compared to the model trained with these new annotations *alongside* the full MELU training dataset (mAP0.5–0.95 of 0.90).

### 3.8 Summary of model results

Table 6 lists the four model assessment measures for all key models in this analysis. Note that ‘swing tag’ is excluded for all outputs in Phases 2 and 3. The measures for models including ‘swing tag’, and only for ‘institutional label’, are included in Tables A2 and A3 respectively.

**TABLE 6:** Four key measures for major model runs [excluding ‘swing tag’ for Phase 2 and 3].

Measures at ‘BEST’ EPOCH				
Phase 1				
MELU‐trained model				All components
	Precision	Recall	mAP0.5	mAP0.5–0.95
Initial	0.976	0.974	0.980	0.837
Revised	0.983	0.969	0.979	0.847

Phase 2									
Validation of *revised* MELU model against Dillen benchmark dataset								Excluding swing tag	
51	Precision	Recall	mAP0.5	mAP0.5–0.95	20	Precision	Recall	mAP0.5	mAP0.5–0.95
B	0.626	0.529	0.566	0.488	B	0.628	0.549	0.564	0.497
BM	0.605	0.682	0.690	0.557	BM	0.656	0.714	0.713	0.581
BR	0.400	0.487	0.487	0.404	BR	0.548	0.480	0.461	0.377
E	0.632	0.659	0.682	0.552	E	0.669	0.592	0.675	0.542
H	0.335	0.434	0.449	0.282	H	0.290	0.458	0.409	0.281
K	0.585	0.557	0.553	0.456	K	0.584	0.521	0.561	0.477
L	0.544	0.555	0.533	0.405	L	0.544	0.606	0.581	0.442
P	0.593	0.600	0.594	0.464	P	0.871	0.537	0.580	0.485
TU	0.362	0.666	0.522	0.364	TU	0.829	0.375	0.624	0.418
All	0.569	0.517	0.521	0.400	All	–	–	–	–

Phase 3														
Retraining: *revised* MELU training dataset + Dillen image‐annotations													Excluding swing tag	
+10	Precision	Recall	mAP0.5	mAP0.5–0.95	+20	Precision	Recall	mAP0.5	mAP0.5–0.95	+30	Precision	Recall	mAP0.5	mAP0.5–0.95
B	0.833	0.740	0.825	0.654	B	0.822	0.831	0.839	0.701	B	0.815	0.841	0.868	0.680
BM	0.886	0.892	0.916	0.695	BM	0.870	0.883	0.915	0.704	BM	0.855	0.876	0.916	0.716
BR	0.852	0.753	0.857	0.676	BR	0.845	0.884	0.863	0.689	BR	0.799	0.799	0.799	0.799
E	0.867	0.862	0.900	0.702	E	0.867	0.948	0.940	0.734	E	0.853	0.922	0.928	0.730
H	0.871	0.869	0.873	0.628	H	0.876	0.884	0.896	0.657	H	0.922	0.881	0.913	0.660
K	0.837	0.714	0.825	0.651	K	0.790	0.826	0.866	0.674	K	0.871	0.797	0.855	0.678
L	0.857	0.833	0.856	0.684	L	0.849	0.892	0.896	0.690	L	0.867	0.882	0.918	0.731
P	0.769	0.870	0.872	0.667	P	0.789	0.768	0.831	0.629	P	0.832	0.838	0.873	0.688
TU	0.896	0.826	0.839	0.651	TU	0.890	0.900	0.922	0.774	TU	0.958	0.923	0.964	0.781
All	0.853	0.843	0.878	0.689	All	0.883	0.867	0.897	0.710	All	0.900	0.824	0.895	0.716

Retraining: *only* Dillen image‐annotations													Excluding swing tag	
10	Precision	Recall	mAP0.5	mAP0.5–0.95	20	Precision	Recall	mAP0.5	mAP0.5–0.95	30	Precision	Recall	mAP0.5	mAP0.5–0.95
All—set 1	0.855	0.829	0.880	0.682	All	0.858	0.879	0.902	0.707	All	0.876	0.868	0.898	0.720
All—set 2	0.854	0.817	0.865	0.665										
All—set 3	0.871	0.819	0.871	0.665										
All—set 4	0.860	0.789	0.857	0.660										
All—set 5	0.768	0.877	0.866	0.674										
Average	0.842	0.826	0.868	0.669										

## 4 DISCUSSION

The above results of this study, as will be explored in more detail in this section, demonstrate that an effective object detection model has been built to identify components of SSDIs. While trained on MELU digitised images, it is shown to be reasonably transferrable to other herbaria SSDIs. The predictive accuracy has been further improved by retraining the MELU model with new image‐annotations.

### 4.1 Phase 1: MELU annotations

On average there were 5.6 annotated components per MELU SSDI (Table 1). Almost all SSDIs have ‘swatch’ and ‘scale’. Of SSDIs without an ‘institutional label’, these had one of the three MELU digitisation labels. Approximately 28% of the annotated MELU SSDIs have one or more taxon annotations and just over 30% have handwriting present on the specimen—this information alone informs prioritisation of future steps to read data from these SSDI components.

As is standard in curation protocols, institutional and annotation labels were consistently placed in the lower right corner of the specimen sheet (Figure 4). This reflects that many of the MELU SSDIs annotated for this research had been remounted prior to digitisation, with consistent instructions for the positioning of components.

### 4.2 Value of annotation task

The initial image annotation work represents the largest resource spent in time and labour for a project such as this. The quality of the trained object detection model relies on the quality of the initial image annotation work, and furthermore, the annotation process provides important visual information for the analyst that can be applied to contextualise model predictive successes and failures. Analysing the annotations extracted from CVAT enabled quality control prior to model training, saving time and compute resources. During the annotation work, types of institutional label, annotation label and stamp were documented: this identified at least 40 different layouts for institutional labels, over 30 different taxon annotation labels and around 15 stamp types. This label diversity, even within a single collection, presents a significant challenge for training an object detection model.

### 4.3 Phase 1: The MELU‐trained sheet‐component model

The first research question—*can a model be built to separately identify labels, handwriting and other original information, taxon annotation labels and other components of a specimen sheet image?* – can be answered positively. A model has been created that identifies the sheet components for MELU herbarium specimens. The key predictive power measures (overall mAP0.5–0.95 of 0.847; Figure 5) indicate that this model can effectively be applied to other MELU SSDIs. The most reliably predicted components (ordered by mAP0.5–0.95) are: ‘swatch’ (0.990), ‘full database label’ (0.985), ‘institutional label’ (0.970), ‘scale’ (0.925), ‘stamp’ (0.891), ‘database label’ (0.887, 16% of background FP), ‘annotation label’ (0.878, 18% of background FP), ‘small database label’ (0.858, only 56 annotation instances), ‘swing tag’ (0.818), ‘handwritten data’ (0.589) and ‘number’ (0.530). These last two components are the most variable in the presentation. As the overall model measures are arithmetic averages of the component results, a poorly predicted component with a small number of annotations—here ‘small database label’—has a potential to unevenly bias the overall model result, and in future, such small‐count components would be excluded.

Moving to the second research question—*how many images must be annotated to train an effective model?* Referring to Figure 6 (left), and comparing against the full MELU training dataset (size of 3371), the testing indicated that (a) the smaller the training dataset the lower the average mAP0.5–0.95, which translates to a lower predictive power of the model, (b) the smaller training datasets had more variability in this measure, which translates to more variable predictive power of the resulting model and (c) the higher variability of models trained on datasets smaller than the validation dataset (i.e. 250, 500, 750) suggests that training datasets should be at least as large as the validation dataset. Additionally, the larger the training dataset the earlier the model reaches stability (right, Figure 6), indicating the larger training datasets could produce a reliable model with fewer than 200 epochs.

For the components of most interest for data collection—the ‘institutional label’ and ‘annotation label—the latter is better identified in training datasets with more than 2000 images, and the former is well predicted across all (Figure 7). While a limitation of this testing regime was that the same sized validation dataset was used throughout (1000 image‐annotations), results indicate that a reasonable model could also be built using training datasets of 2000 and 2500 image‐annotations.

Heterogeneity among SSDI components and their layout—as herbaria include specimens from various collectors, collections and over different curation eras—means that substantial training and validation datasets are required to ensure all possible specimen sheet component types are trained for and validated against. Even a validation dataset of 23% (1000 of 4371 for the full MELU model) demonstrated variability in model outcomes when four different mutually exclusive validation datasets were applied (Figure 6, left). Therefore, the number of images to be annotated and used to train a sheet‐component object detection model is contingent on the uniformity of the SSDI components, with fewer images needed for training a model where these are consistent in appearance and position. Further, these results show the allocation of image‐annotations to the training or validation datasets will also affect the resulting model, therefore care must be taken to randomise, or carefully manually select, so as not to bias prevalence in either dataset and to check that all components are equally represented.

### 4.4 Phase 2: Applying to new images without training

The concentrated locations of institutional and annotation labels noted in the MELU SSDIs (Figure 4) were also seen in the SSDIs from the Dillen et al. (2019) study (Figure 8). While the lower left corner of the specimen sheet is also commonly used for both label types, there is more variability in overall placements (as expected, given these are results across different herbaria) particularly for ‘annotation label’.

When the *revised* MELU‐trained sheet‐component object detection model was applied to the benchmark image‐annotations (without retraining the model) the results varied across the nine herbaria and uphold the basic object detection tenet that a model works best with components close to those it was trained on. Referring to Figure 9, the transferability of ‘institutional label’ and ‘annotation label’ was satisfying, though it was noted that some ‘annotation labels’ are little more than free‐hand text on unformatted paper and the MELU‐trained model confused these with ‘original (handwritten) data’. ‘Swatch’ and ‘scale’ were not as generalisable as anticipated; while some benchmark SSDIs included some elements similar to those used by MELU, many have versions not seen by the MELU‐trained model. The barcode element is not present on any MELU image, and for the benchmark dataset was identified as ‘number’ to test how the trained model would handle the new information. As expected, it was initially poorly identified.

It should also be noted that the SSDIs selected from the benchmark dataset, and annotated for this investigation, were chosen without consideration of how the specimens were ordered in that dataset. While all of the specimens met the requirements of the Dillen et al. (2019) study, specimens from each participating herbarium varied significantly, for example, in the label or stamp types present, the placement of the labels or stamps, as well as in the format (typed or handwritten) and arrangement of the data on the label. Therefore, a different selection of SSDIs from the benchmark dataset will result in different model outcomes.

That said, it can be asserted that the *revised* MELU‐trained sheet‐component object detection model could be directly applied to new SSDIs not from MELU to identify and locate sheet components and would predict reasonably well, particularly for the ‘institutional label’. As for all models though, targeted retraining could be conducted to improve outcomes (covered in the next section).

### 4.5 Phase 3: Applying to new images or components with retraining

Adding new image‐annotations to the full MELU training dataset resulted, in most cases, in better predictions than using the untrained MELU model alone. The differences between the two validation sets (*pale* and *dark grey* bars) are hypothesised to be due to the different individual SSDIs in the two validation datasets, again reinforcing the impact of bias of images included/excluded in this dataset. The results that went against expectations are likely due to the individual features of the SSDIs included in each of the small additional training datasets and how they align with the features of SSDIs in the validation datasets. It is also noticeable that only Berlin (benchmark herbarium ‘B’) retrained with 20 additional image‐annotations produces a mAP0.5–0.95 close to the original MELU model for this component. The ‘scale’ improvements demonstrate the improvement that minor retraining has on predictions (Figure 11, right) even more clearly. Adding as few as 10 new image‐annotations has raised mAP0.5–0.95 for Berlin by 0.42, Meise (BR) by 0.71 and Helsinki (H) by 0.89. Adding 20 and 30 shows variable improvement across the herbaria.

However, the results of the next set of tests indicate that the additional time required to train new image‐annotations *alongside* MELU training data is not balanced by improvements in outcome (Table 4). Training on *only* 30 new image‐annotations per herbaria took 3.5 hours, whereas training *alongside* MELU training image‐annotations data took 9 hours. Comparing the models that tested training on 20 or 30 image‐annotations, it appears that training *alongside* MELU training data is only slightly beneficial, though more so for some components (e.g. ‘swing tag’, which is barely present in the benchmarks SSDIs).

The test results, therefore, provide an outline for answering the third research question—*how many new annotated images are needed to retrain a model for a new feature or collection (with different types of labels for example)?* As might be expected, the more image‐annotations used to retrain a model the better the outcome. However, where there is consistency in the appearance and location on the SSDI of a new type of a known component, such as the ‘scale’ in Helsinki SSDIs, as few as 10 new image‐annotations added to the training dataset appeared sufficient for reasonable prediction (Figure 11). However, if the SSDI component is visually diverse, or the component not placed in the same location on all SSDIs—e.g. the barcodes coded as a ‘number’, while all looking the same, were not in the same location on all SSDIs within a set (likely due to being applied at digitisation and their position dictated by all other components present)—30 new image‐annotations is insufficient for retraining for accurate prediction.

Additionally, when retraining the MELU model for new components in the benchmark dataset, only minor improvement was observed when adding the new image‐annotation to the full MELU training dataset (of 3371). Therefore, if compute, time and memory space are limited, it is recommended to: (i) annotate 30 SSDIs if the component is stable, or annotate each different type 20 times; (ii) start with the MELU‐model weights for YOLOv5; (iii) retrain (for as few as 50 epochs) *only* on the new image‐annotations. This provides a starting point only; the more variable the appearance and location of the component, the more image‐annotations are generally required.

### 4.6 Further work

The research team has incorporated the MELU‐trained sheet‐component model described here into a pipeline named ‘Hespi’ (*He*rbarium *S*pecimen *Pi*peline) (R. Turnbull, K. M. Thompson, E. Fitzgerald, & J. L. Birch, unpublished data). This pipeline takes the institutional label identified by the model described here, crops it out, identifies each text element in the label (using a separate ‘label‐text’ object detection model), extracts the text and applies OCR to output all data available on the institutional label.

## 5 CONCLUSION

This research successfully built an object detection model to identify institutional labels, handwriting and other original information, taxon annotation labels and other components of herbarium specimen sheet digital images. The application of YOLOv5 to annotations of digital images from the MELU digitised collection (3371 for training, 1000 for validation) was a straightforward process. The resulting model demonstrates good predictive outcomes for many sheet components for MELU, though handwritten data proved understandably problematic.

Applying the MELU‐trained sheet‐component model to another set of digitised herbarium images reinforced the basic object detection tenet that a model works best with components close to those it was trained on. The prediction of the component of key interest for data extraction, ‘institutional label’, was solid; and that for ‘annotation label’ was better than anticipated. The MELU model may be applied to images from other herbaria unaltered, though retraining will improve predictivity. Such retraining could be run solely on the new image‐annotations, and where components look the same and are consistently placed as few as 10 new annotations may be sufficient. However, all testing undertaken during this investigation repeatedly emphasised a fundamental point: the more heterogeneous the components and their location on the specimen sheet, the more digital images must be annotated to (re)train a sufficient model.

The model built here will be incorporated into the digitisation protocol at MELU as part of the application of the Hespi pipeline. Further, such machine‐driven component identification, particularly when focussed on labels and integrated with text reading, has the potential for application to many kinds of collections that have initiatives focussed on the digitisation of data stored on pro‐forma object or specimen labels.

## AUTHOR CONTRIBUTIONS

**Karen M. Thompson:** Conceptualization (equal); data curation (equal); formal analysis (equal); investigation (lead); methodology (equal); project administration (equal); validation (lead); visualization (lead); writing – original draft (lead); writing – review and editing (lead). **Robert Turnbull:** Conceptualization (lead); data curation (equal); formal analysis (equal); investigation (equal); methodology (lead); project administration (equal); resources (lead); software (lead); supervision (lead); writing – review and editing (supporting). **Emily Fitzgerald:** Writing – review and editing (supporting). **Joanne L. Birch:** Conceptualization (equal); funding acquisition (lead); investigation (equal); methodology (supporting); project administration (equal); resources (equal); supervision (equal); validation (supporting); visualization (supporting); writing – original draft (equal); writing – review and editing (equal).

## Data Availability

With the intent to contribute to the research of other herbaria and supporting research teams, the following assets and outputs from this research are made available on the condition of (a) full citation and (b) open and like‐for‐like sharing of resulting research: (1) Annotations for MELU SSDIs (images may be accessed online via the collection portal); https://doi.org/10.26188/23597013. (2) MELU‐trained sheet‐component object detection model weights (for application in YOLOv5); https://doi.org/10.26188/23597034.

## APPENDIX 1

**TABLE A1:** Training and validation sets from MELU annotations; size of training image‐annotation dataset in the left‐most column.

	Sample dataset GROUP name					
	Initial training	Train 10set	Fold1	Fold2	Fold3	Fold4
	SET 1	SET 2	SET 3			
	A		B	C	D	E
Validation (1000)	Randomly selected		Mutually exclusive – a specimen sheet will be in only one of these sets			
250	Training‐250	Sample_250_1	Sample‐250‐1	Same naming convention as fold 1	Same naming convention as fold 1	Same naming convention as fold 1
		Sample_250_2	Sample‐250‐2			
		…	…			
		Sample_250_10	Sample‐250‐10			
500	Training‐500	sample_500_1	sample‐500‐1			
		… etc	… etc			
750	Training‐750	Sample_750_1	Sample‐750‐1			
		… etc	… etc			
1000	Training‐1000	Sample_1000_1	Sample‐1000‐1			
		… etc	… etc			
1500	Training‐1500	Sample_1500_1	Sample‐1500–1			
		… etc	… etc			
2000	Training‐2000	–	Sample‐2000‐1			
			Sample‐2000‐2			
2500	Training‐2500	–	Sample‐2500–1			
			Sample‐2500‐2			
3371 (max)	Training‐3371	–	Training 3371	Training 3371	Training 3371	Training 3371
Note 1	Samples not nested					
Note 2	–	Samples randomly selected from [full‐validation dataset], with replacement				
		(i.e. a specimen sheet may be in more than one dataset)				
Note 3	–	Insufficient variability to make a 10‐set for sizes 2000 and 2500	Investigated 2 samples for sizes 2000 and 2500, to test variability.			
Dataset count	Validation: 1	Validation: ‐	Validation: 1	Validation: 1	Validation: 1	Validation: 1
(total = 283)	Training sets: 8	Training sets: 50	Training sets: 55	Training sets: 55	Training sets: 55	Training sets: 55

**TABLE A2:** Four key measures for major model runs, Phase 2 and 3 including ‘swing tag’.

MEASURES at ‘BEST’ EPOCH—for all components combined									
Phase 2									
Validation of *revised* MELU‐model against Dillen benchmark dataset								Including swing tag	
51	Precision	Recall	mAP0.5	mAP0.5–0.95	20	Precision	Recall	mAP0.5	mAP0.5–0.95
B	0.654	0.526	0.557	0.483	B	0.628	0.549	0.564	0.497
BM	0.655	0.719	0.729	0.566	BM	0.656	0.714	0.713	0.581
BR	0.475	0.426	0.433	0.358	BR	0.605	0.42	0.428	0.344
E	0.677	0.767	0.699	0.541	E	0.709	0.602	0.681	0.532
H	0.333	0.505	0.517	0.340	H	0.290	0.458	0.409	0.281
K	0.594	0.543	0.545	0.447	K	0.636	0.581	0.616	0.517
L	0.572	0.552	0.551	0.405	L	0.576	0.630	0.600	0.420
P	0.644	0.588	0.582	0.450	P	0.871	0.537	0.580	0.485
TU	0.362	0.666	0.522	0.364	TU	0.829	0.375	0.624	0.418
All	0.596	0.521	0.531	0.400	All	–	–	–	–

Phase 3														
Retraining: revised MELU training dataset + Dillen image‐annotations													Including swing tag	
+10	Precision	Recall	mAP0.5	mAP0.5–0.95	+20	Precision	Recall	mAP0.5	mAP0.5–0.95	+30	Precision	Recall	mAP0.5	mAP0.5–0.95
B	0.833	0.74	0.825	0.654	B	0.822	0.831	0.839	0.701	B	0.815	0.841	0.868	0.680
BM	0.886	0.892	0.916	0.695	BM	0.870	0.883	0.915	0.704	BM	0.855	0.876	0.916	0.716
BR	0.871	0.784	0.875	0.691	BR	0.842	0.899	0.879	0.715	BR	0.824	0.867	0.895	0.721
E	0.882	0.879	0.912	0.697	E	0.884	0.949	0.947	0.716	E	0.871	0.918	0.936	0.720
H	0.871	0.869	0.873	0.628	H	0.876	0.884	0.896	0.657	H	0.922	0.881	0.913	0.660
K	0.858	0.750	0.846	0.669	K	0.816	0.848	0.882	0.677	K	0.863	0.822	0.872	0.706
L	0.856	0.829	0.849	0.650	L	0.868	0.856	0.900	0.665	L	0.883	0.847	0.898	0.684
P	0.769	0.870	0.872	0.667	P	0.789	0.768	0.831	0.629	P	0.832	0.838	0.873	0.688
TU	0.896	0.826	0.838	0.651	TU	0.890	0.900	0.922	0.774	TU	0.958	0.923	0.964	0.781
All	0.846	0.793	0.834	0.647	All	0.873	0.838	0.873	0.682	All	0.898	0.834	0.899	0.702

Retraining: *only* Dillen image‐annotations													Including swing tag	
10	Precision	Recall	mAP0.5	mAP0.5–0.95	20	Precision	Recall	mAP0.5	mAP0.5–0.95	30	Precision	Recall	mAP0.5	mAP0.5–0.95
All—set 1	0.857	0.786	0.841	0.635	All	0.865	0.840	0.866	0.659	All	0.850	0.847	0.887	0.690
All—set 2	0.872	0.767	0.853	0.631										
All—set 3	0.867	0.796	0.853	0.634										
All—set 4	0.857	0.766	0.843	0.628										
All—set 5	0.779	0.851	0.852	0.636										
Average	0.846	0.793	0.848	0.633										

**TABLE A3:** Four key measures for major model runs, for ‘institutional label’ only.

Measures at ‘Best’ epoch—Institutional label only				
Phase 1				
MELU‐trained model				
	Precision	Recall	mAP0.5	mAP0.5–0.95
Revised	0.999	0.992	0.995	0.970

Phase 2									
Validation of *revised* MELU‐model against Dillen benchmark dataset									
51	Precision	Recall	mAP0.5	mAP0.5–0.95	20	Precision	Recall	mAP0.5	mAP0.5–0.95
B	0.730	0.846	0.924	0.857	B	0.778	0.971	0.978	0.931
BM	0.798	0.894	0.933	0.889	BM	0.788	0.938	0.950	0.903
BR	0.870	0.902	0.930	0.834	BR	0.916	0.886	0.914	0.809
E	0.861	0.746	0.910	0.802	E	1.000	0.775	0.960	0.814
H	0.670	0.906	0.933	0.891	H	0.531	0.864	0.879	0.849
K	0.843	0.667	0.796	0.667	K	1.000	0.694	0.823	0.749
L	0.593	0.882	0.779	0.702	L	0.556	0.895	0.847	0.766
P	0.880	0.800	0.899	0.763	P	0.851	0.634	0.867	0.762
TU	0.398	0.981	0.930	0.681	TU	0.942	0.772	0.907	0.706
All	0.776	0.836	0.890	0.771	All	–	–	–	–

Phase 3														
Retraining: r*evised* MELU training dataset + Dillen image‐annotations														
+10	Precision	Recall	mAP0.5	mAP0.5–0.95	+20	Precision	Recall	mAP0.5	mAP0.5–0.95	+30	Precision	Recall	mAP0.5	mAP0.5–0.95
B	0.791	1.000	0.973	0.918	B	0.775	1.000	0.995	0.968	B	0.725	1.000	0.978	0.900
BM	0.792	0.938	0.924	0.877	BM	0.770	0.938	0.949	0.916	BM	0.737	0.938	0.961	0.917
BR	0.889	0.868	0.914	0.856	BR	0.847	0.865	0.919	0.859	BR	0.836	1.000	0.991	0.934
E	1.000	0.870	0.995	0.853	E	0.984	1.000	0.995	0.900	E	0.952	1.000	0.995	0.909
H	0.832	0.904	0.923	0.887	H	0.909	0.909	0.937	0.899	H	0.953	0.915	0.979	0.934
K	0.989	0.783	0.963	0.876	K	0.967	0.783	0.937	0.858	K	1.000	0.757	0.864	0.798
L	0.818	0.944	0.934	0.853	L	0.771	0.947	0.874	0.743	L	0.837	0.947	0.954	0.904
P	0.801	0.926	0.928	0.839	P	0.742	0.963	0.921	0.854	P	0.790	0.963	0.933	0.872
TU	0.950	0.910	0.988	0.815	TU	0.934	1.000	0.995	0.908	TU	0.945	1.000	0.995	0.912
All	0.844	0.932	0.952	0.873	All	0.908	0.929	0.967	0.900	All	0.904	0.941	0.966	0.907

Retraining: *only* Dillen image‐annotations														
10	Precision	Recall	mAP0.5	mAP0.5–0.95	20	Precision	Recall	mAP0.5	mAP0.5–0.95	30	Precision	Recall	mAP0.5	mAP0.5–0.95
All—set 1	0.901	0.920	0.970	0.892	All	0.894	0.926	0.969	0.902	All	0.885	0.951	0.966	0.910
All—set 2	0.890	0.925	0.954	0.858										
All—set 3	0.897	0.913	0.964	0.867										
All—set 4	0.887	0.917	0.952	0.867										
All—set 5	0.850	0.929	0.959	0.868										
Average	0.885	0.921	0.960	0.870										
